# Cesium Carbonate-Catalyzed α-Phenylchalcogenation of Carbonyl Compounds with Diphenyl Dichalcogenide

**DOI:** 10.3390/molecules14093367

**Published:** 2009-09-02

**Authors:** Yutaka Nishiyama, Yuya Koguma, Toshimasa Tanaka, Rui Umeda

**Affiliations:** Faculty of Chemistry, Materials and Bioengineering, Kansai University, Suita, Osaka 564-8680, Japan

**Keywords:** cesium carbonate, α-phenylchalcogenation, carbonyl compounds, diphenyl dichalcogenide

## Abstract

It was found that cesium carbonate has a unique catalytic ability on the reaction of carbonyl compounds with diphenyl diselenide to give the corresponding α*-*phenylseleno carbonyl compounds in moderate to good yields. Similarly, the α-phenylthiolation of carbonyl compounds with diphenyl disulfide was promoted by the cesium carbonate catalyst.

## Introduction

**Scheme 1:**
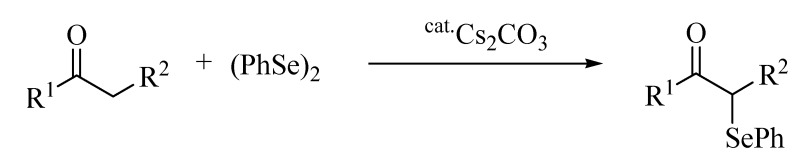
Cesium carbonate-catalyzed α-phenylselenation of carbonyl compounds with diphenyl diselenide.

α*-*Phenylselenocarbonyl compounds are useful synthetic intermediates in organic synthesis [1,2,3,4,5] and much effort is being devoted to accomplish the synthesis of these compounds. There are many reports on the preparation of α*-*phenylselenocarbonyl compounds: (i) electrophilic reaction with PhSeX, PhSeX_3_, PhSeO_2_CCF_3_ or phenylselenium cation (PhSe^+^), which was generated *in situ* from the treatment of diphenyl diselenide with SeO_2_, benzeneseleninic anhydride, chloramine-T, or Et_4_NBr or MgBr_2_ under electrolysis [6,7,8,9,10,11,12,13,14,15,16,17,18,19,20,21]; (ii) nucleophilic displacement of α-halocarbonyl compounds with sodium and lithium phenylselenolate [22]; (iii) insertion of carbene into carbon-selenium bond of selenol esters [23]; (iv) palladium-catalyzed coupling of phenyl tributylstannyl selenide and α-halocarbonyl compounds [24] and (v) organocatalyst-catalyzed reaction of carbonyl compounds with *N*-(phenylseleno)phthalimide [25,26,27,28]. However, many of these synthetic methods suffer from the the problems of handling the selenium reagents used as selenium sources because of their instability towards air and moisture, acidic or basic reaction conditions, and the use of stoichiometric amounts of acid or base. Thus, the development of new synthetic methods using stable selenium reagents under mild conditions would have significant synthetic value. We now discovered that a cesium salt acts as a catalyst on the α-phenylselenation of carbonyl compounds with diphenyl diselenide giving the corresponding α*-*phenylselenocarbonyl compounds in moderate to good yields (Scheme 1).

## Results and Discussion

When 5-nonanone (**1a**; 0.30 mmol) was allowed to react with an equivalent amount of diphenyl diselenide (0.30 mmol) in the presence of a catalytic amount of cesium carbonate (0.05 mmol, 17 mol%) in DMA solvent at 70 °C for 5 h, the α-phenylselenation of **1a** smoothly proceeded to give 4-phenylseleno-5-nonanone (**2a**) in 0.03 mmol yield (entry 2 in Table 1). In the absence of the cesium salt, **2a** was not formed and the starting materials were recovered (entry 1). 

**Table 1:** Reaction of 5-nonanone with diphenyl diselenide. ^a^

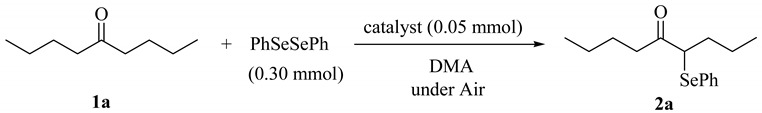					
**entry**	**1a/mmol**	**temp/ °C**	**time/h**	**catalyst**	**yield/mmol ^b^**
1	0.30	70	5	none	trace
2	0.30	70	5	Cs_2_CO_3_	0.03
3	0.90	70	5	Cs_2_CO_3_	0.03
4	1.50	70	5	Cs_2_CO_3_	0.05
5	3.00	70	5	Cs_2_CO_3_	0.07
6	3.00	70	10	Cs_2_CO_3_	0.23
7	3.00	100	5	Cs_2_CO_3_	0.59
8 ^c^	3.00	100	5	Cs_2_CO_3_	0.27
9	3.00	100	5	CsF	0.42
10	3.00	100	5	CsCl	0.06
11	3.00	100	5	CsBr	0.08
12	3.00	100	5	CsI	0.02
13	3.00	100	5	K_2_CO_3_	0.48
14	3.00	100	5	Na_2_CO_3_	0.17

^a^ *Reaction conditions*: PhSeSePh (0.30 mmol), catalyst (0.05 mmol), and DMA (2.5 mL); ^b^ GC yield. ^c^ Under a nitrogen atmosphere.

Increasing the amount of **1a**, extending the reaction time, and elevating the reaction temperature led to the increase the yield of **2a** (entries 2-7). When diphenyl diselenide was treated with ten equivalent amounts of **1a** (3.00 mmol) under air at 100 °C for 5 h, **2a** was obtained in 0.59 mmol yield, along with a formation of a small amount of 4,4-bisphenylseleno-5-nonanone (entry 7). It is interested to note that both phenylseleno groups on diphenyl diselenide are efficiently utilized on the reaction. When the reaction was carried out under a nitrogen atmosphere, the yield of **2a** was decreased (entry 8). Similarly, in the presence of a catalytic amount of cesium fluoride, the α-phenylselenation of **1a** occurred to give **2a** in 0.42 mmol yield (entry 9). In contrast to cesium carbonate and fluoride, in the case of the other cesium salts, such as cesium chloride, bromide and iodide, the α-phenylselenation did not proceed (entries 10-12). In the presence of another alkaline metal carbonates, the yields of **2a** decreased slightly (entries 13 and 14) [29].

To determine the scope and limitation of the cesium salt-catalyzed α-phenylselenation of carbonyl compounds with diphenyl diselenide, various carbonyl compounds were allowed to react with diphenyl diselenide in the presence of a catalytic amount of cesium carbonate (17 mol%) and these results are shown in Table 2. The treatment of cyclohexanone with diphenyl diselenide under the same reaction conditions as that of 5-nonanone afforded 2-phenylselenocyclohexanone in 0.43 mmol yield (entry 2). For the reaction of the acetophenone and propiophenone, which have an aromatic ring adjacent to the carbonyl group, the α-phenylselenation of these compounds took place smoothly to give the corresponding α-phenylselenoacetophenone and -propiophenone in 0.51 and 0.59 mmol yields, respectively (entries 4 and 8).

4’-Methyl-, 4’-methoxy-, and 4’-chloro-α-phenylselenoacetophenone were prepared by the cesium carbonate-catalyzed reaction of the corresponding acetophenone derivatives with diphenyl diselenide in moderate to good yields (entries 5-7). When an unsymmetrical dialkyl ketone such as 2-octanone was treated with diphenyl diselenide in the presence of cesium carbonate, the phenylselenation of methylene carbon predominantly proceeded to give 3-phenylseleno-2-octanone in 0.41 mmol yield with the formation of 1-phenylseleno-2-octanone (0.17 mmol) (entry 3). In the case of hexanal, the yield of α-phenylselenoaldehyde was low (0.13 mmol) under the same reaction conditions as that of 5-nonanone, owing to the formation of a complex reaction mixture that included the aldol type products; however, the α-phenylselenated product yield was improved, when the reaction was carried out under the lower reaction temperature (65 °C) (entry 9).

Next, the cesium carbonate-catalyzed reaction of carbonyl compounds with diphenyl disulfide was examined (Table 3). When 5-nonanone (3.0 mmol) was allowed to react with diphenyl disulfide (1.0 mmol) in the presence amount of cesium carbonate at 100 °C for 5 h, the α-phenylthiolation of 5-nonanone was successfully occurred and 4-phenylthio-5-nonanone was obtained in 0.44 mmol yield (entry 1). The treatment of cyclohexanone or acetophenone with diphenyl disulfide also afforded the α-phenylthioketone in 0.60 or 0.51 mmol yields (entries 2 and 3).

**Table 2:** Cesium carbonate-catalyzed α-phenylselenation of carbonyl compounds with diphenyl diselenides. ^a^

entry	carbonyl compound	product	yield/mmol ^b^
1	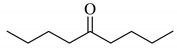	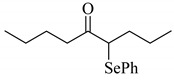	0.59
		**2a**	
2	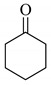	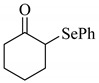	0.43
		**2b**	
3	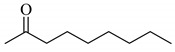	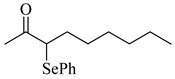	0.41
		**2c**	
		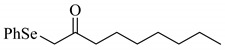	0.17
		**2c’**	
	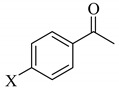	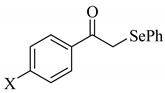	
4	X = H	**2d**; X = H	0.51
5	X = CH_3_	**2e**; X = CH_3_	0.54
6	X = OCH_3_	**2f**; X = OCH_3_	0.48
7	X = Cl	**2g**; X = Cl	0.56
8	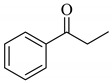	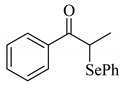	0.59
		**2h**	
9	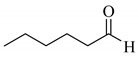	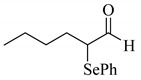	0.43
		**2i**	

^a^ Reaction conditions: PhSeSePh (0.30 mmol), carbonyl compound (3.00 mmol), Cs_2_CO_3_ (0.05 mmol), and DMA (2.5 mL) under air at 100 °C for 5 h. ^b^ GC yield.

**Table 3:** Cesium carbonate-catalyzed α-phenylthiolation of carbonyl compounds with diphenyl disulfide. ^a^

entry	carbonyl compound	product	yield/mmol ^b^
1	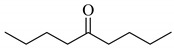	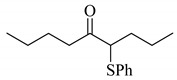	0.44
		**3a**	
2	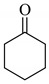	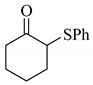	0.60
		**3b**	
3	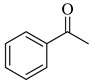	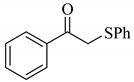	0.51
		**3c**	

^a^ Reaction conditions: PhSSPh (1.0 mmol), carbonyl compound (3.0 mmol), Cs_2_CO_3_ (0.2 mmol), and DMA (2.5 mL) under air at 100 °C for 5 h. ^b^ GC yield.

**Scheme 2:**
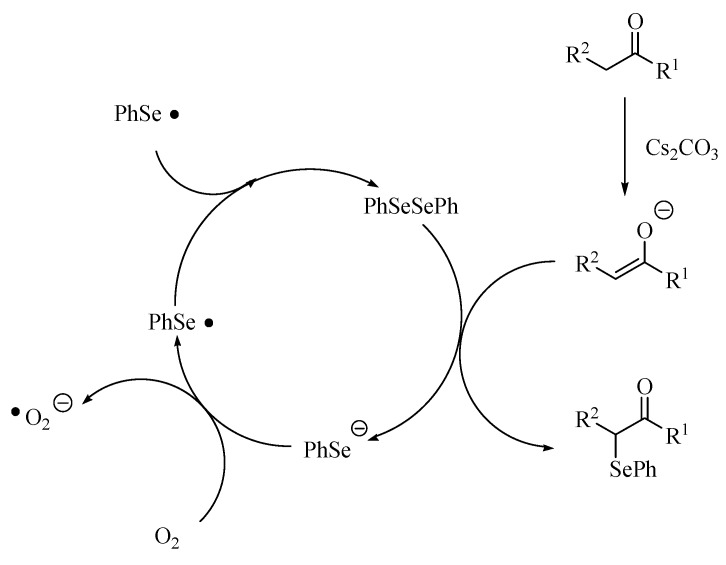
A plausible reaction path.

Although we cannot explain the reaction pathway of the carbonyl compounds with diphenyl diselenide in the presence of cesium carbonate catalyst in detail, one possible reaction pathway is shown in Scheme 2. The first step of the reaction is the generation of the enolate anion by the deprotonation of carbonyl compound with the cesium salt [30]. The enolate anion was trapped with diselenide to form α-phenylselenocarbonyl compounds, and the phenylselenolate [31]. When the reaction was carried out under air, the product yield was higher than when performed under a nitrogen atmosphere (entries 7 and 8 in Table 1). From the result, it was suggested that phenylselenolate was oxidized by oxygen to form phenylseleno radical and subsequently dimerized giving the diphenyl diselenide.

In summary, we found a unique catalytic ability of cesium carbonate in the reaction of carbonyl compounds with diphenyl dichalogenides. The α-phenylchalcogenation of carbonyl compounds with diphenyl dichalcogenides, such as the diselenide or disulfide in the presence of cesium carbonate catalyst efficiently proceeded to give the α-phenylchalcogenocarbonyl compounds in moderate to good yields.

## Experimental

### General

The ^1^H- and ^13^C-NMR spectra were recorded on a JEOL AL400 spectrometer using CDCl_3_ as the solvent with tetramethylsilane as the internal standard. The IR spectra were recorded with a PerkinElmer FT-IR2000 spectrometer. Gas chromatography (GC) was performed on a Shimadzu GC-14B gas chromatograph using a flame-ionizing detector-equipped instrument and a CBP1 capillary column (0.25 mm × 1200 mm). Preparative HPLC separation was undertaken with a JAI LC-908 chromatograph using 600-mm × 20-mm JAIGEL-1H and 2H GPC columns with CHCl_3_ as an eluent.

### Reagents

Diphenyl disulfide, carbonyl compounds, cesium salts, and alkaline metal salts were purchased as high grade products, and used without purfication. Diphenyl diselenide was synthesized as described in the literature [22]. The solvents were purified before use by the usual methods.

### General procedure for cesium salt-catalyzed reaction of carbonyl componds with diphenyl diselenide

A DMA (2.5 mL) solution of diphenyl diselenide (94 mg, 0.30 mmol), carbonyl compound (3.00 mmol), and cesium carbonate (16 mg, 0.05 mmol) was stirred at 100 °C for 5 h under air. After the reaction was complete, H_2_O was added to the reaction mixture and extracted with diisopropyl ether. The organic layer was dried over MgSO_4_. The resulting mixture was filtered, and the filtrate was concentrated. Purification of the residue by HPLC afforded the corresponding α-phenylselenated product. The product was characterized by comparing its spectral date with those of authentic samples (**2a** [32], **2b** [27], **2d** [24], **2e** [24], **2f** [24], **2g** [24], **2h** [24], and **2i** [27]). The structures of the product were assigned by their ^1^H- and ^13^C-NMR, IR, and Mass spectra.

*Mixture* **2c** *and* **2c’** (**2c:2c’** = 41:17): ^1^H-NMR (CDCl_3_) δ 7.53-7.46 (m, 2H), 7.33-7.20 (m, 3H), 3.63 (t, *J* = 7.4 Hz, 0.70H), 3.56 (s, 0.61H), 2.53 (t. *J* = 7.4 Hz, 0.61H), 2.26 (s, 2.1H), 1.87-1.76 (m, 0.70H), 1.73-1.62 (m, 0.70H), 1.58-1.48 (m, 0.70H), 1.47-1.38 (m, 0.70H), 1.37-1.14 (m, 7.2H), 0.87 (t, *J* = 6.8 Hz, 3H); ^13^C-NMR (CDCl_3_) δ 205.56, 204.04, 135.36, 135.31, 133.01, 129.06, 128.95, 128.41, 127.60, 127.18, 52.29, 40.51, 35.80, 31.46, 31.40, 30.14, 28.86, 28.81, 28.74, 27.85, 27.19, 23.80, 22.41, 22.35, 13.90, 13.86; IR (NaCl) 3388, 3058, 2925, 2855, 1703, 1578, 1477, 1438, 1355, 1066, 1022, 1000, 739, 691, 670 cm^-1^; MS m/z 298.

### General procedure for cesium salt-catalyzed reaction of carbonyl compounds with diphenyl disulfide

A DMA (2.5 mL) solution of diphenyl disulfide (218 mg, 1.0 mmol), carbonyl compound (3.0 mmol), and cesium carbonate (64 mg, 0.2 mmol) was stirred at 100 °C for 5 h under air. After the reaction was complete, H_2_O was added to the reaction mixture and extracted with diisopropyl ether. The organic layer was dried over MgSO_4_. The resulting mixture was filtered, and the filtrate was concentrated. Purification of the residue by HPLC afforded the corresponding α-phenylselenated product. The product was characterized by comparing its spectral date with those of authentic samples (**3a** [32], **3b** [33], and **3c** [34]). The structures of the product were assigned by their ^1^H- and ^13^C-NMR, IR, and Mass spectra.
